# Case report: Intracoelomic neoplastic mass of undetermined origin in an Asia minor spur-thighed tortoise (*Testudo graeca ibera*)

**DOI:** 10.3389/fvets.2023.1265034

**Published:** 2023-11-14

**Authors:** Camille François, Clément Paillusseau, Stefano Bagatella, Francesco Origgi, Lionel Schilliger

**Affiliations:** ^1^SpéNac Referral Center, Paris, France; ^2^Department of Infectious Diseases and Pathobiology, Vetsuisse Faculty, Institute of Animal Pathology (ITPA), University of Bern, Bern, Switzerland

**Keywords:** case report, spur-thighed tortoise, *Testudo graeca ibera*, plastrotomy, neoplasia

## Abstract

This article describes the diagnostic, treatment and attempted characterization of a neoplasia of undetermined origin in a Asia minor spur-thighed tortoise. A 21-year-old male Asia minor spur-thighed tortoise (*Testudo graeca ibera*) was admitted for a 4-month history of diarrhea, and a 2-month history of anorexia and lethargy. Physical examination revealed a firm midcoelomic mass in the right prefemoral fossa. Blood biochemistry indicated hypocalcemia and mild elevation of aspartate aminotransferase. Supportive care was administered in the form of heating, baths, and calcium injections. Ultrasound examination of the coelomic cavity revealed a 6-cm diameter, highly vascularized mass with liver-like echogenicity. Neoplasia was suspected, and endoscopy was performed, revealing a brown circumscribed mass with smooth edges. Surgical removal of the mass was evaluated by CT scan and achieved via a plastrotomy; however, the patient died 1 day post-surgery. The mass was located on the dorsal right side of the coelomic cavity in the anatomic location of the right testicle. Histopathology revealed neoplastic cells organized in packets supported by fibrous septa. Neoplastic cells showed moderate and inconsistent positive immunohistochemical labeling for S100 and NSE, and negative immunohistochemical labeling for pan-cytokeratin, vimentin, CD3, CD79a, chromogranin A, and synaptophysin. The prominent histological and anatomical characteristics of the mass indicated a possible testicular or neuroendocrine (e.g., adrenal gland) origin. Due to inconclusive immunohistochemical profiles and poorly differentiated neoplastic cells, only a final diagnosis of intracoelomic malignant tumor of undetermined origin could be established. This case underscores the difficulties encountered in achieving definitive diagnoses of neoplastic diseases in reptile medicine.

## 1. Introduction

The Asia minor Spur-thighed Tortoise (*Testudo graeca ibera*) is a subspecies of the spur-thighed tortoise (Testudo graeca) with a range extending from the Balkans to the Middle East (1). This species is frequently presented in veterinary practice by virtue of its popularity as a pet. Neoplasia, however, is thought to be rare in pet tortoises (1.2%), especially compared to other reptiles such as snakes (2.9%) and lizards (3%) (2). Tumors of the reproductive system have been reported in tortoises, including in a Spur-Thighed Tortoise (*T. graeca*) and in several desert tortoises (Gopherus agassizi) (3–5). Their diagnosis, however, remains infrequent (4) and challenging due to the vast number of species within the reptilian order and the limited number of documented cases. Specific characterization of reptilian tumors using immunohistochemistry is further complicated by the lack of species-specific antigens (4). In this article, we describe a neoplastic mass with unusual morphological, histological, and immunohistochemical features. By providing a comprehensive description of the diagnostic and treatment approach, we highlight the challenges of immunohistochemical characterization of tumors in chelonians.

## 2. Case description

A 21-year-old male Asia minor spur-thighed tortoise (*Testudo graeca ibera*) was admitted with a 4-month history of diarrhea, and a 2-month history of weight loss and dysorexia. The patient had not suffered from any medical condition before this episode. The tortoise was captive bred and lived alone in an indoor terrarium. The owner reported a diurnal temperature of 25–29°C (77–84°F) under the basking spot and a nocturnal temperature of 20°C (68°F). The tortoise was fed daily with a large variety of green vegetables. The owner provided neither UVB exposure nor calcium supplementation. The tortoise was initially treated by another veterinarian with fenbendazole (Panacur, 25 mg/mL, MSD Santé Animale, Beaucouze, France, 50 mg/kg PO SID; three treatments) for suspected gastrointestinal helminths. Following 15 days without clinical improvement, the tortoise was admitted for additional investigation.

On physical examination, the tortoise was lethargic, emaciated, and moderately dehydrated (based on salivary film thickness and mucous membrane tackiness). It displayed lethargy and reduced mobility. Cloacal and buccal mucosae were normal. Respiratory rate was 10 breaths per minute [reference range = 11–27 in Hermann’s tortoise (*Testudo hermanni*) and marginated tortoise (*Testudo marginata*)] at room temperature (~25.2°C) (6). Heart rate was 72 beats per minute [reference range = 34–70 in T. hermanni and Horsfield’s tortoise (*Testudo horsfieldii syn. Agrionemys horsfieldii*)] (6, 7). Coelomic palpation through the right prefemoral fossa revealed a firm mass that did not elicit a pain response and could not be attributed to a specific anatomical structure. On direct fecal examination, no intestinal parasites could be seen. A green coloration of the urine was consistent with biliverdinuria.

## 3. Diagnostic assessment and therapeutic intervention

**Figure 1:**
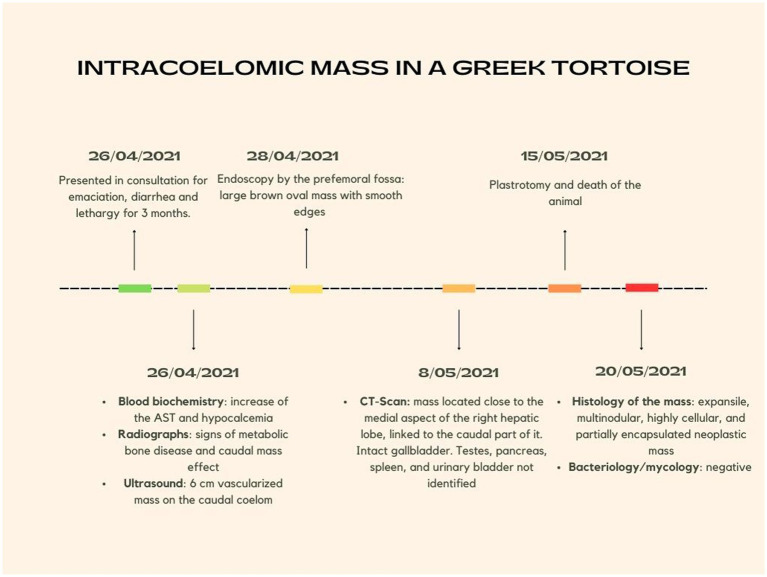
Timeline summarizing the chronology of the episode of care.

Because of the absence of a reference range for blood biochemistry in the spur-thighed tortoise, those established for the closely related Hermann’s tortoise were used (Figure 1). Blood biochemistry revealed increased AST (449 U/L; reference range = 0–359) and hypocalcemia (1 mg/dL; reference range = 4.8–20.5) (8). Other biochemical parameters were unremarkable (Table 1). Blood smear cytology did not reveal any abnormalities but confirmed the dehydration status of the animal.

**Table 1:** Immunohistochemical labeling of chelonian tissues using the antibody panel adapted for tumoral characterization, and the corresponding immunohistochemical labeling on mammal tissue controls.

Marker	Tortoise tissues^*^	Mammalian control tissues
*Vimentin*	Negative (all tissues)	Salivary gland and skin: dermal fibroblasts, diffuse cytoplasmic positivity.
*CD3*	Lymphocytes in spleen (and interstitial lymphocytes in various other organs): diffuse cytoplasmic positivity	Lymphnode: lymphocytes, diffuse cytoplasmic positivity
*NSE*	Negative (all tissues)	Adrenal gland: diffuse cytoplasmic positivity (++ medullary cells)
*CD79a*	Smooth muscle in all tissues (++ muscularis of arteries, gastrointestinal tract, epidydimis): diffuse to granular cytoplasmic positivity	Lymphnode: lymphocytes, diffuse cytoplasmic positivity
*S100*	Brain (neuropil): diffuse cytoplasmic positivity. Smooth muscle in all tissues (++ muscularis of arteries, gastrointestinal tract, epidydimis): diffuse to granular cytoplasmic positivity. Adrenal gland (interrenal cells): patchy, membranous to irregularly cytoplasmic	Adrenal gland: diffuse cytoplasmic positivity (both cortical and medullary cells)
*Chromogranin A*	Negative (all tissues)	Adrenal gland: rare cells in cortex and medulla show diffuse, irregular cytoplasmic staining
*Synaptophysin*	Negative (all tissues)	Adrenal gland (medullary cells): diffuse, cytoplasmic staining

^*^The following tissues from a male, 25 years-old Testudo hermanni were analyzed: testis (with epididymis), adrenal gland, kidney, pancreas, brain, spleen, large intestine, skeletal muscle, tongue.

The animal was immediately hospitalized and received supportive care including heating, bathing, force-feeding (Emeraid Intensive care Herbivore, EmerAid LLC, Cornell, IL, U.S.A, 15 mL/kg PO), and injections of calcium gluconate (Theracalcium 32,5 mg/mL, Vetoquinol, Lure, France, 100 mg/kg IM). Within 15 days, the patient was adequately rehydrated and blood calcium was normalized, although it continued displaying lethargic behavior.

Radiographs showed a loss of contrast in the ventral coelomic cavity, a ventral mass effect, widespread decrease in skeletal opacity, and cortical bone thinning. Ultrasound examination using a micro-convex probe (8Mhz) through the right and left prefemoral fossae revealed a circumscribed mass caudal to the right liver lobe. The mass was round and circumscribed (6 cm diameter). The appearance was heterogeneous and highly vascularized, with a liver-like echogenicity. A mild effusion was visible between the two liver lobes. The right kidney, part of the liver, and the testes could not be assessed. Endoscopy via a right prefemoral approach was performed, using a rigid endoscope under general anesthesia. After induction (Domitor, medetomidine 0,85 mg/mL, Vetoquinol S.A., France 0.08 mg/kg IM; Clorketam, ketamine 100 mg/mL, Vetoquinol S.A, France, 10 mg/kg IM), the patient was intubated and maintained on isoflurane (Isovet, isoflurane 1,000 mg/g, Osalia, France, 2–4%) with 1.5 L/min oxygen flow. Breathing was mechanically assisted and a capnography monitor was used. On endoscopy, a large brown oval mass with smooth edges was visible. Five 3 mm biopsies were taken for bacteriological and mycological analysis, which returned negative results. Additional samples were analyzed histologically and revealed features compatible with neoplasia (histological features are described later in the manuscript).

A CT scan was performed 10 days after the endoscopy to evaluate the precise location of the mass and prepare for surgical removal, on the unrestrained patient. The effusion surrounding the mass reduced intracoelomic contrast. Air was located in the dorsocaudal part of the coelomic cavity, which was attributed to the endoscopic procedure a few days prior. Some mineralization was observed in the left ventral part of the coelomic cavity, which was suspected to originate from leakage of the gallbladder under the compressive effect of the mass. A highly vascularized tissular heterogeneous mass (4.5 × 7.6 × 5.5 cm) was confirmed after injection of contrast material (Omnipaque, 350 mg/mL, GE Healthcare SAS, Vélizy Villacoublay, France, 3 mL/kg IV). The mass was located close to the medial aspect of the right hepatic lobe and linked to the caudal part of it. The gallbladder was intact, whereas the testes, pancreas, spleen, and urinary bladder could not be visualized due to the space-occupying nature of the mass.

**Figure 2:**
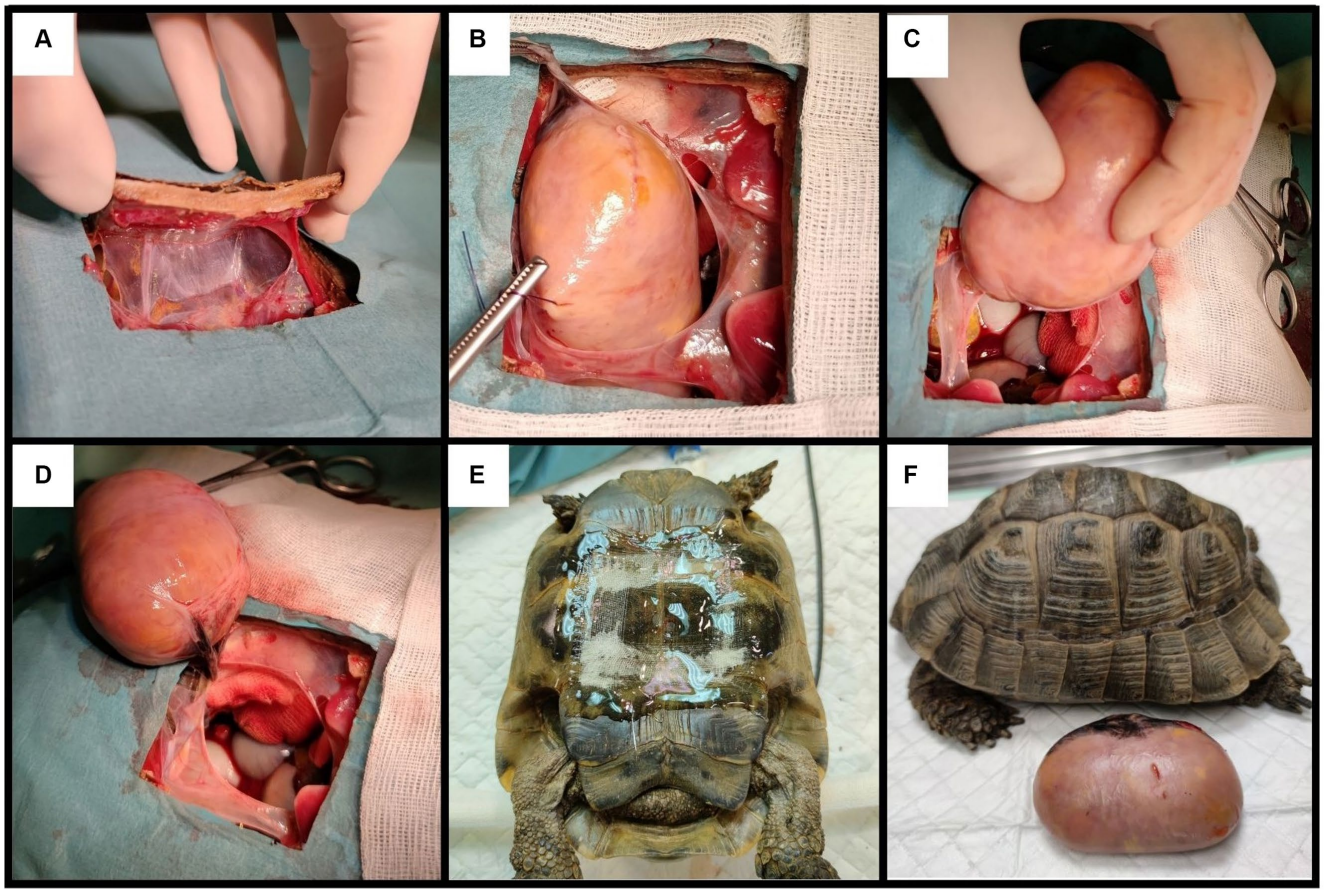
Steps of the plastrotomy: **(A)** creation of the bone flap; **(B,C)** removal of the mass using stay sutures; **(D)** mass connected to the coelomic cavity by a single fibrous ligament, suspected to be the mesorchium; **(E)** closing the bone flap using fiberglass and epoxy; **(F)** patient and mass post-surgery.

Surgery was elected as the best possible treatment. Due to the size and location of the mass, its removal could not be attempted through the prefemoral fossa; plastrotomy was elected instead (Figure 2). Pre-surgical analgesia was provided with tramadol chlorhydrate (Tralieve 50 mg/mL, Dechra, Montigny le Bretonneux, France, 10 mg/kg IM) 1 hour prior to induction. The patient was anesthetized using the same protocol as described above. A surgical window of approximately 10 × 6 cm was drawn above the mass, between the middle of the pectoral and the abdominal scales, as determined by the CT longitudinal reconstruction. A conventional plastrotomy was performed using a high speed sterilized 2 cm diameter disk (Dremel 4,000, Bosh, Germany) (9). After reflecting the bone flap, the mass was elevated from the coelom using stay sutures. The mass was located in the dorsal part of the right side of the coelomic cavity, close to the anatomic location of the right testis, and was attached to the dorsal coelom via a fibrous band resembling the mesorchium. This pedicle was ligated using absorbable multifilament suture material (Vicryl 3–0, Coveto, Paris, France). No other dissection or hemostasis was needed. The right testis could not be found in the coelomic cavity. The coelomic membrane was closed routinely, and the bone flap was fixed with fiberglass held in place with epoxy resin. Following surgery, the animal received tramadol (10 mg/kg IM SID), meloxicam (Meloxidyl, 5 mg/mL, Ceva Santé Animale, Libourne, France, 0.2 mg/kg IM SID) and oxytetracycline (Oxytetracycline 5%, 50 mg/mL, Vetoquinol, Paris, France, 10 mg/kg IM SID).

The tortoise made a full recovery from anesthesia (i.e., was able to breathe without ventilation within 50 min post-surgery) but died 24 h after surgery. Permission to perform a full necropsy was denied by the owners.

**Figure 3:**
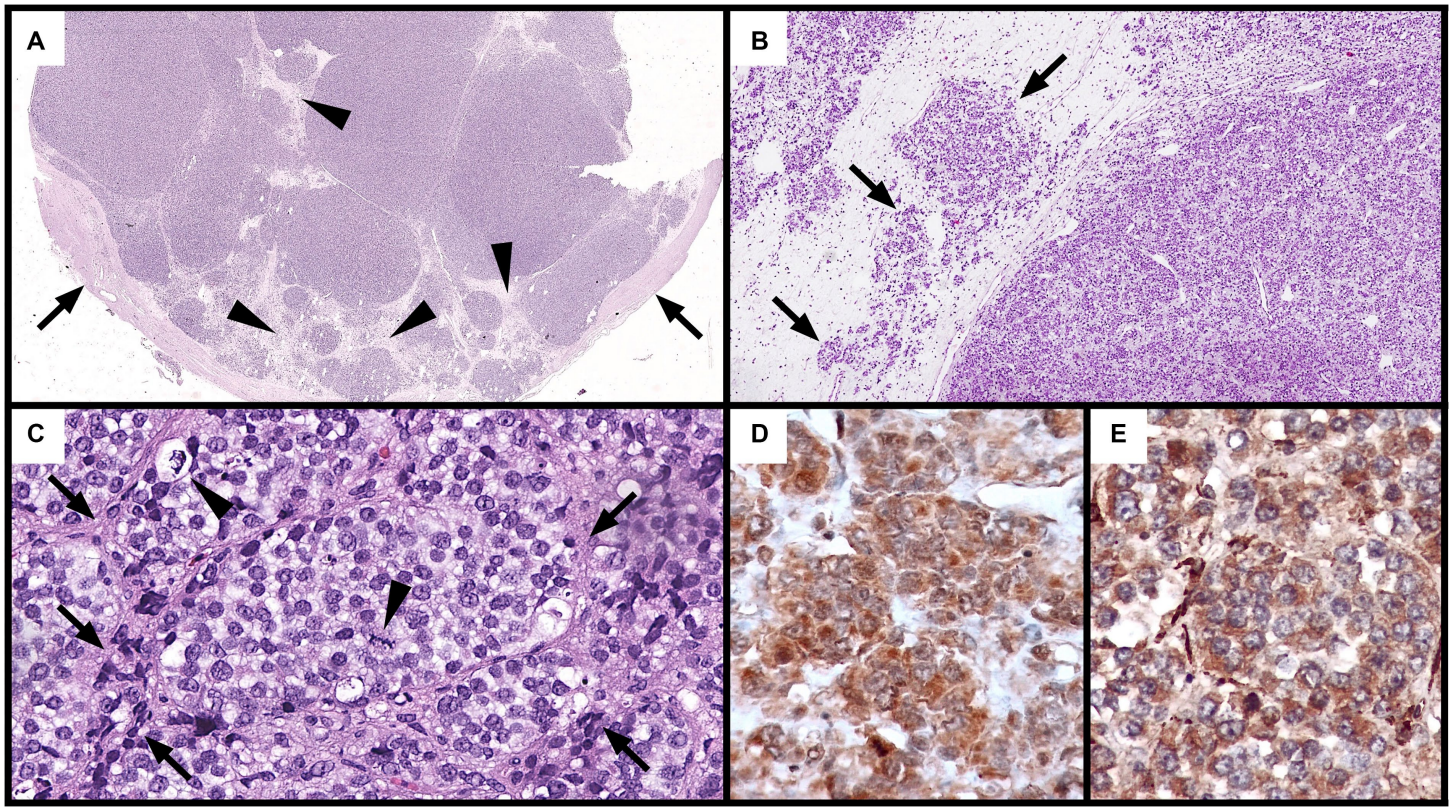
Histological and immunohistochemical features of the neoplastic mass **(A–C)**: hematoxylin and eosin. **(A)** Multiple, highly cellular nodular aggregates of neoplastic cells separated by connective tissue septa (arrows) and partially surrounded by a thick, fibrous capsule (Cp: capsule) (5x). **(B)** Loosely arranged packets of neoplastic cells (right) separated by thick connective septa (left). Local infiltration of neoplastic cells into these septa is observed (arrows) (50x). **(C)** Packets of round to oval neoplastic cells with ill-distinct borders and vacuolated cytoplasm encased by thin fibrovascular stroma (arrows). Two mitotic figures (arrowheads) are observed (400x). **(D,E)** Positive cytoplasmic immunohistochemical staining of neoplastic cells for S100 **(D)** and NSE **(E)** from the first immunohistochemistry round (400x).

The mass was submitted for histopathological examination to an external laboratory. Histopathological examination revealed an expansile, multinodular, highly cellular, and partially encapsulated neoplastic mass (Figure 3A). Neoplastic cells were organized in loosely arranged packets intermixed with scant fibrovascular stroma (Figures 3B,C). They were separated by thick, mildly cellular septa consisting of few elongated or stellate cells, and few small blood vessels which were immersed in a loose, poorly fibrillary matrix. Neoplastic cells were multifocally embedded into these septa (Figure 3B). Neoplastic cells were polygonal to oval, had ill-defined cytoplasmic borders, and scant eosinophilic to moderate cytoplasm or contained numerous, variably sized, optically empty cytoplasmic vacuoles. Nuclei were round to oval, moderately eccentric, with coarsely stippled chromatin and contained up to two recognizable nucleoli (Figure 3C). Anisocytosis and anisokaryosis were marked, and 48 mitotic figures were seen in 10 HPF. Multifocally, single heterophils were scattered in the neoplastic mass. The neoplasm was partially enclosed by a thick, fibrous capsule (Figure 3A) containing numerous vessels lined by endothelium, which were only occasionally filled by erythrocytes. The capsule was multifocally invaded by small clusters of neoplastic cells. Adjacent to the capsule were bands of connective tissue containing melanomacrophages and non-neoplastic structures (suspected to be the ductus deferens and epididymis) consisting of ducts composed of cuboidal, simple to pseudostratified ciliated epithelium, as well as a moderate number of non-neoplastic ducts composed of a simple columnar epithelium.

Immunohistochemical staining showed diffuse cytoplasmic positive immunolabeling of neoplastic cells for S100 (calcium binding protein) (Figure 3D) and NSE (neuron specific enolase, Figure 3E). Neoplastic cells were negative for pan-cytokeratin, vimentin (mesenchymal cell marker), CD3 (T-cell marker), CD79a (B-cell marker), and markers for neuroendocrine tumors (chromogranin A and synaptophysin). Positive controls from mammalian tissues were used for all markers except for cytokeratin, in which positive staining of the peritumoral glands and ducts acted as positive control. Histological slides were submitted to the Vetsuisse Faculty of Bern for a second diagnostic opinion. Histological features of the tumor were confirmed and the previously performed immunohistochemical panel was validated on chelonian tissues (Table 1). Staining of the tumor for S100 and NSE, however, proved negative (not shown).

The anatomical location of the mass, the absence of a recognizable right testis, the presence of a fibrous ligament suggestive of the mesorchium, and epithelial lined tubular elements consistent with testicular adnexa suggested a tumor of testicular origin. The main histological features of the mass were, however, less conclusive and more consistent with a poorly differentiated neoplasm with features resembling a neuroendocrine tumor of unknown origin (packets of cells separated by connective tissue septa and fine blood vessels). The poor differentiation of the neoplastic cells, along with their marked anisocytosis, anisokaryosis, and high mitotic rate were suggestive of a malignant neoplasia. It cannot be fully excluded that the mass originated from an endocrine organ adjacent to the testis, such as the adrenal gland. The immunohistochemical panel selected for this investigation (CD3, CD79a, pan-cytokeratin, vimentin, chromogranin A, synaptophysin, S100, and NSE) aimed to establish a conclusive diagnosis, or at the very least, narrow the differential diagnosis. Unfortunately, a species-matched positive control was only available for the pan-cytokeratin (patient peritumoral ducts); immunohistochemical characterization of this tumor is therefore limited without species-specific validated reagents and matched tissues. Taken together, the mass was most consistent with either a poorly differentiated malignant testicular tumor or a malignant neuroendocrine neoplasm.

## 4. Discussion

Seminomas and Leydig cell tumors have been reported in chelonians (3–5), while Sertoli cell tumors have been reported exclusively in snakes (2, 10, 11). In these cases, histological and cellular patterns were inconsistent with those observed in the patient and led to the exclusion of a seminoma (cords or sheets of large, round to polygonal cells with large nuclei with prominent nucleoli) and Leydig cell tumor (acini or sheets of large, round to polygonal cells with abundant cytoplasm and homogeneous nuclei, and low mitotic rate). A malignant Sertoli cell tumor was not completely ruled out given the presence of prominent connective septa, cytoplasmic vacuolation, and positive immunolabeling with NSE on the first round of immunohistochemical characterization—a marker expressed in Sertoli cell tumors in both mammalian and non-mammalian species (12, 13). A Sertoli cell tumor, could, however, not be confirmed because immunolabeling with vimentin —a mesenchymal marker expressed in testicular tumors in mammals—was inconclusive (Meuten et al., 2016), as it was the case in normal testis of lizards of the Leiosauridae family (15). Second, antibodies specific to Sertoli cells (e.g., anti-Müllerian hormone and inhibin α) that have successfully been used in non-mammalian species were not available to the authors (13, 16). Third, transmission electron microscopy for the identification of ultrastructural features typical of Sertoli cells, such as Charcot-Böttcher crystals (13), could not be performed. On the other hand, some features (notably the histological pattern of neuroendocrine packeting) indicated a malignant neuroendocrine tumor of undetermined origin which may have originated from the adrenal gland (given the prominently vacuolated cytoplasm of neoplastic cells suggestive of interrenal cells) or, alternatively, from the diffuse endocrine tissue (4). Interrenal neoplasms have not been reported to occur in chelonians but have been observed in various snake species and a green iguana (4). No attempts at characterizing these tumors through immunohistochemistry have been made. Chromogranin, synaptophysin, and NSE are considered to be the most reliable markers for neuroendocrine tumors (17). NSE immunolabeling has been demonstrated in a neuroendocrine carcinoma in a bearded dragon (17), however, another report on neuroendocrine tumors in bearded dragons found that NSE immunolabeling was not detected, and positivity for synaptophysin (40% of tumors) and chromogranin (20% of tumors) was inconsistent (18). The high inconsistency in immunolabeling could explain the negative stain for chromogranin and synaptophysin observed in the patient’s tumor which, together with the lack of ultrastructural characterization of neuroendocrine features (particularly neuroendocrine granules) prevented a conclusive diagnosis of a neuroendocrine neoplasm (17, 18). While S100 immunolabeling has been observed in mammalian neuroendocrine tumors, its broad cellular expression hampers its diagnostic specificity (19). In reptiles, S100 has been previously used to successfully label chromatophoromas and various mesenchymal tumors (fibroma, myxoma, myxosarcoma) in different species (20), and a peripheral nerve sheath tumor in a bearded dragon (21), which are not morphologically compatible with this case. The diagnostic significance of S100 immunolabeling in this case, together with the negative tumoral staining on the second round of immunohistochemical characterization, is therefore questionable and does not help to identify the potential cellular origin of this neoplasm. The lack of repeatability of S100 and NSE staining is most likely attributable to variations in immunohistochemical processing between the two institutions.

The lack of CD3 expression, a marker successfully used in reptiles, excludes a T-cell lymphoma, while a B-cell neoplasm can be excluded based on morphological features, rather than lack of immunostaining for CD79a, which has given conflicting results when used in reptiles (4). Furthermore, cytokeratin negativity rules out an epithelial origin or component of the mass. Cytokeratins have previously been detected in numerous reptile neoplasms of epithelial origin, including a scent gland adenoma, ovarian cystadenocarcinoma, esophageal adenocarcinoma, pancreatic ductular adenocarcinoma, renal adenocarcinoma, and periocular adenocarcinoma (22–27).

In conclusion, this case highlights the need to establish and validate adequate immunohistochemical markers and species-specific tissue controls for characterizing tumors in chelonians. Despite a comprehensive morphological characterization, the neoplastic origin of this mass could not be determined, although circumstantial and histopathological evidence suggest a possible testicular or neuroendocrine nature. The clinical history, findings, and investigations were insufficient in narrowing down the differential diagnoses, whereas imaging played an important role in detecting the mass and providing information for defining the therapeutic approach.

Despite significant advancements in reptile medicine over the past 30 years, there is still much to be accomplished in addressing challenging diagnostic cases, with the case described herein serving as a typical example.

## Data availability statement

The original contributions presented in the study are included in the article/supplementary material, further inquiries can be directed to the corresponding author.

## Ethics statement

The supplementary examinations and proposed interventions were always aimed at promoting the animal's recovery and the owner provided oral consent at each step. No experimental treatments were tested on the animal, and for these evident reasons, an ethics committee or written owner consent was not required.

## Author contributions

CF: Investigation, Methodology, Writing – original draft. CP: Investigation, Methodology, Writing – original draft. SB: Investigation, Methodology, Writing – original draft. FO: Investigation, Supervision, Writing – original draft. LS: Writing – original draft.
